# Effects of SMILE on intraocular pressure and corneal biomechanics: a systematic review and meta-analysis

**DOI:** 10.1007/s10792-026-04194-5

**Published:** 2026-08-24

**Authors:** Stamatios Lampsas, Gerasimia-Marina Chardalia, Efthymios Karmiris, George D. Kymionis, Irini Chatziralli

**Affiliations:** 1 Second Department of Ophthalmology, School of Medicine, National and Kapodistrian University of Athens, Attikon University Hospital, 1, Rimini Street, 12462 Haidari, Athens, Greece; 2 First Department of Ophthalmology, Medical School, “G. Gennimatas” Hospital, National and Kapodistrian University of Athens, 11528 Athens, Greece

**Keywords:** SMILE, Intraocular pressure, Corneal biomechanics, Corneal hysteresis, Refractive surgery

## Abstract

**Purpose:**

Small incision lenticule extraction (SMILE) is a popular flapless refractive procedure for myopia and myopic astigmatism, although postoperative corneal biomechanical changes may affect intraocular pressure measurements and glaucoma assessment. This systematic review and meta-analysis aimed to quantify the postoperative underestimation of IOP and changes in corneal biomechanical parameters following SMILE.

**Materials and methods:**

A comprehensive literature search of PubMed, Cochrane Library, ScienceDirect, and Scopus databases was performed up to April 30, 2026, according to PRISMA guidelines. Observational studies reporting preoperative and postoperative IOP measurements after SMILE using Goldmann applanation tonometry (GAT), Ocular Response Analyzer (ORA), or Corvis ST were included. Secondary outcomes included changes in corneal hysteresis (CH) and corneal resistance factor (CRF).

**Results:**

Seventeen studies involving 1177 eyes were analyzed. Postoperative IOP was significantly reduced across all tonometric modalities. The pooled mean reduction was greatest with GAT (MD: 3.47 mmHg; 95% CI 2.85–4.10), followed by ORA (MD: 2.50 mmHg; 95% CI 2.06–2.94) and Corvis ST (MD: 1.52 mmHg; 95% CI 0.62–2.42) (all* p* < 0.001). Significant postoperative reductions were observed in CH (MD: 1.84 mmHg) and CRF (MD: 3.01 mmHg), indicating decreased corneal biomechanical stability after SMILE.

**Conclusions:**

SMILE leads to significant underestimation of IOP measurements and measurable biomechanical weakening of the cornea. Clinicians should interpret postoperative IOP values cautiously, even when using biomechanically corrected tonometric devices.

**Supplementary Information:**

The online version contains supplementary material available at 10.1007/s10792-026-04194-5.

## Introduction

Small incision lenticule extraction (SMILE), first described by Sekundo et al. in 2010, is a flapless lamellar refractive procedure [1]. The technique involves the creation of an intrastromal lenticule and its extraction through a small incision using a femtosecond laser. It is primarily indicated for the correction of myopia and myopic astigmatism. Over the last decade, SMILE has gained increasing popularity because of the advantages it provides over other refractive techniques, including faster visual recovery, lower incidence of postoperative dry eye symptoms [2–4], as well as avoidance of well-described flap-related complications of LASIK, such as epithelial ingrowth [5,6].

Although SMILE has proven to have a favorable safety and efficacy profile [7,8], interfering with the corneal stroma’s structure may affect postoperative ocular measurements. In this context, IOP is known to be influenced by corneal parameters, including thickness, curvature, and biomechanical indices [9,10]. Furthermore, it must be accurately assessed during ophthalmologic follow-up examinations, especially for glaucoma screening and monitoring. There are many devices (tonometers) used in everyday clinical practice to assess IOP following corneal refractive surgeries, including Goldmann applanation tonometry (GAT), Corvis ST, and the Ocular Response Analyzer (ORA), and their results are variably influenced by corneal alterations. Beyond IOP, detection of potential postoperative changes in the viscoelastic and stiffness-related indices of the cornea, such as Corneal Hysteresis (CH) and Corneal Resistance Factor (CRF) provided by the ORA, may help explain differences in IOP measurements.

Several studies have investigated the effect of SMILE on IOP measurements and corneal biomechanics. However, the magnitude of postoperative IOP reduction varies considerably, mainly because of differences in tonometric devices used, follow-up duration, and corneal biomechanical indices assessed [11,12]. In parallel, although individual studies have also reported SMILE-induced biomechanical changes [13–15], the extent to which these changes may explain the observed decrease in postoperative IOP measurements remains unclear.

Therefore, our meta-analysis aims to evaluate the effect of SMILE on IOP in myopic eyes or eyes with myopic astigmatism. The primary outcome was the postoperative change in measured IOP, analyzed according to the tonometric device used (GAT, ORA and CORVIS ST). Secondary outcomes involved postoperative changes in CH and CRF.

## Materials and methods

### Literature search

The present systematic review and meta-analysis was performed following the methodological framework recommended by the Preferred Reporting Items for Systematic Reviews and Meta-Analyses (PRISMA) guidelines. Our study protocol has been officially registered with PROSPERO under the REGISTER ID: CRD420261394454. A thorough literature search was made in PubMed, Cochrane Library, ScienceDirect and Scopus databases up to 30th April 2026, using search queries addressing the predefined main outcome of our study, focusing on the IOP change after SMILE surgery (Supplementary Table S1). Two researchers (S.L, GM.C.), independently assessed studies’ eligibility based on prespecified protocol criteria. In case of doubtful eligibility, a decision was made through an interactive discussion between the researchers. The reference lists of the retrieved articles were also manually screened, using the “snowball” approach, and any additional studies meeting the eligibility criteria were included.

### Study selection and data extraction

Eligibility criteria comprised the following: i) Patients included in the selected studies had to be adults aged 18 years or older, ii) Peer-reviewed articles had to be written in English or have an abstract in English including the required measurements for the meta-analysis, iii) Studies had to report IOP measurements preoperatively and at least 1 month postoperatively in patients who underwent SMILE surgery for myopia or myopic astigmatism, iv) IOP had to be measured with validated tonometers such as Goldmann applanation tonometry (GAT), Ocular Response Analyzer (ORA) and/or Corneal Visualization Scheimpflug Technology (Corvis ST). For studies using the Ocular Response Analyzer, only corneal-compensated intraocular pressure (IOPcc) measurements were extracted. Original, observational research articles, including retrospective and prospective case series, cohort, and cross-sectional studies, meeting the aforementioned criteria were selected for inclusion. Review articles, preclinical animal studies, studies published in languages other than English and studies including patients treated with SMILE for hyperopia were excluded. In addition, to enhance the accuracy and transparency of the study selection process, we structured our inclusion criteria according to the PICOS framework for systematic reviews and meta-analyses: (i) participants (P)—patients undergone SMILE for correcting myopia or myopic astigmatism; ii) intervention (I)—SMILE surgery with preoperative IOP measurement by GAT, ORA and/or Corvis ST; iii) comparator (C)—postoperative IOP measurement at least one month after SMILE surgery; iv) outcome (O)—mean difference in IOP between preoperative and postoperative measurements; v) study (S)—observational studies. Duplicate records were removed using Zotero software (version 7.0.12), which was also used to facilitate the screening process.

### Quality assessment

The final 17 included studies were evaluated for methodological quality and risk of bias using the Newcastle–Ottawa Scale (NOS) for cohort studies, since all included studies were of single-arm, pre–post observational design and no case–control studies were retrieved. The cohort NOS comprises three primary domains: (i) Selection of the study cohort (0–4 points, covering representativeness of the exposed cohort, selection of the non-exposed cohort, ascertainment of exposure, and demonstration that the outcome of interest was not present at baseline), (ii) Comparability of study groups (0–2 points, awarded based on adjustment for confounders), and (iii) Outcome ascertainment and follow-up (0–3 points, covering assessment of outcome, adequacy of follow-up duration, and completeness of follow-up). However, the assessment was adapted for the current review, as all included studies reported pre–post SMILE data rather than conventional exposed-versus-non-exposed comparisons. For these single-arm designs, evaluation was based on representativeness of the SMILE cohort, ascertainment of the intervention, reliability of outcome measures, data completeness, adequacy of follow-up, and potential unit-of-analysis concerns (i.e., inclusion of both eyes from the same patient without correction for inter-eye correlation). The maximum attainable score remained 9 points, and study quality was classified as poor, fair, or good for scores of 0–3, 4–6, and 7–9, respectively. Two researchers independently assessed the included studies and reached consensus on the final report, which is provided in Supplementary Table S2.

### Statistical analysis

Statistical analyses were performed using the RevMan 5.4 software (The Cochrane Collaboration, Oxford, UK). Continuous outcomes were summarized as mean differences (MD) with corresponding 95% confidence intervals (CI). Pooled estimates were calculated using a random-effects model, given expected clinical and methodological differences across studies. Heterogeneity was assessed using the Cochran’s Q statistic (χ2 testing), with a* p* value of 0.05 considered statistically significant, and quantified using the I2 statistic with values classified into three categories: > 75%, 50–75% and < 50% representing substantial, moderate and low heterogeneity, respectively. Although α = 0.10 is sometimes recommended for Q to compensate for its low power, interpretation of heterogeneity in this review relied primarily on I^2^, for which classification thresholds are conventional and independent of test power. All results are presented in forest plots. Sensitivity analyses applying the leave-one-out approach were performed only for meta-analyses involving more than five studies to ensure results’ reliability. Moreover, publication bias was assessed by visual inspection of funnel plots when appropriate. Egger’s regression test was performed for IOP measurements obtained with the Corvis ST, as this was the only outcome including more than ten studies, using IBM SPSS Statistics version 29.0.2 (IBM Corp., Armonk, NY, USA).

## Results

### Search results

**Fig. 1:**
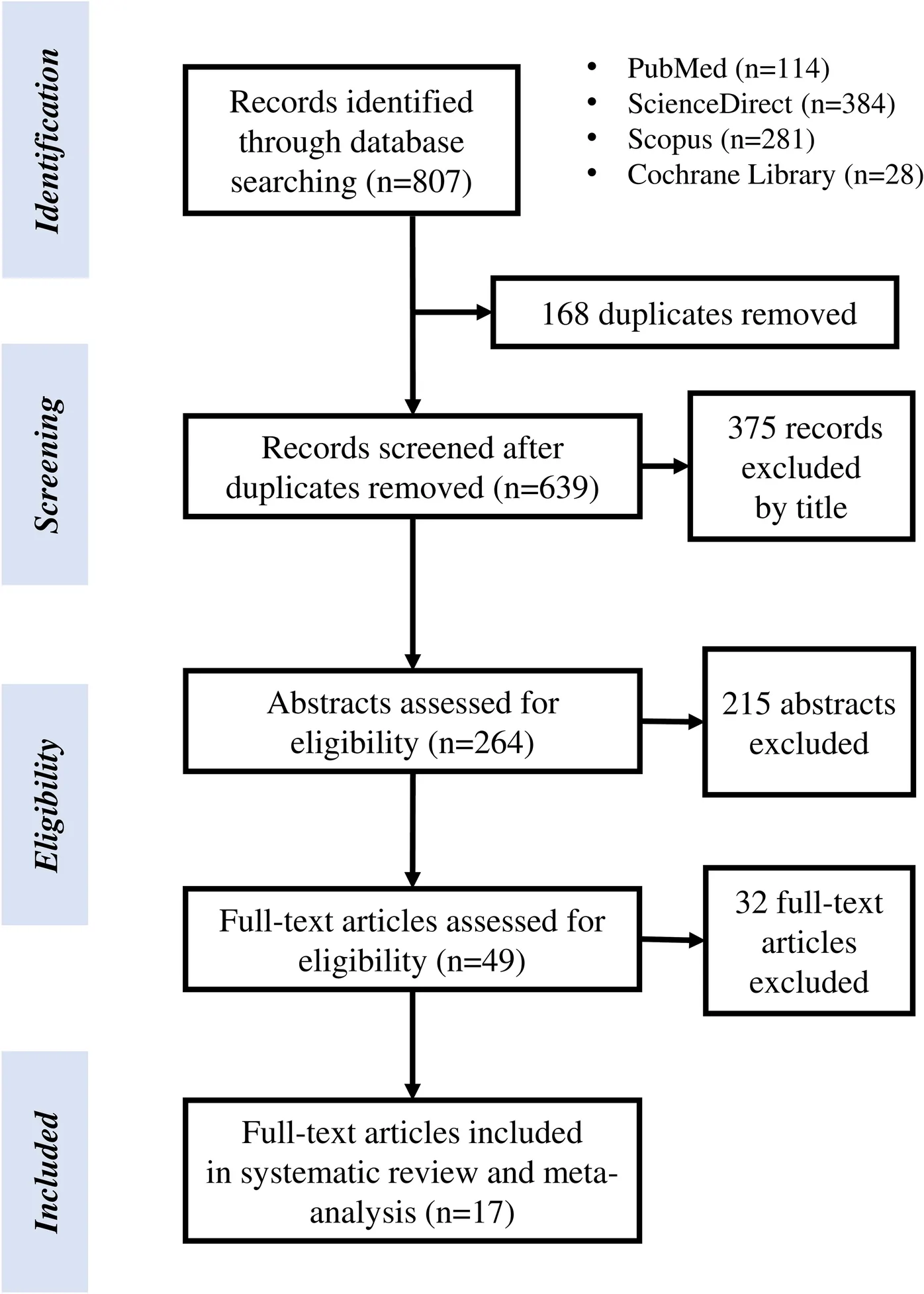
PRISMA flowchart for study selection

Following the removal of duplicates, 807 records were identified and screened at the title level and 264 the abstract level. Of these, 215 were excluded for failing to meet the relevance criteria. The remaining 49 articles underwent full-text evaluation, of which 17 studies satisfied all predefined eligibility criteria and were ultimately included (Fig. 1). A total of 1177 eyes that underwent refractive surgery were included, with a mean patient age of 26.4 ± 6.1 years; 45.7% of the participants were male. Six studies (35%) analyzed one eye per patient, whereas the remaining studies included both eyes or did not explicitly specify the unit of analysis. The main characteristics of the included studies are summarized in Table 1 [11–13,16–29]. The mean duration of follow-up across studies was 3 months.

**Table 1:** Characteristics and findings of the included studies

Study	Type of study	Type of tonometer	Sample size (eyes)	Age (yrs)	Sex (male)	Population	Key findings
Abd El-Fattah et al. [19]	Prospective cohort	Corvis ST	30	26.4	50%	60 eyes with myopia <− 8D and myopic astigmatism <− 4D (30 eyes assigned for LASIK and 30 eyes for SMILE)	bIOP significantly decreased 6 months postoperatively group by 2.52 ± 1.389 mmHg (*p* < 0.001), but there was no significant association with CCT
Cao et al. [17]	Prospective cohort	Corvis ST	40	26.8	40%	80 eyes non-randomly, equally assigned to SMILE (40 eyes) and FS-LASIK (40 eyes)	SMILE may impair corneal biomechanics more than LASIK, although both procedures similarly reduce biomechanics when the same CCT is removed. Postoperatively, Corvis ST-evaluated IOP is more accurately than non-contact tonometry
Chen et al. [18]	Prospective cohort	GAT, Corvis ST, ORA	50	27.1	38%	144 myopic eyes assigned for FS-LASIK (50 eyes), SMILE (50 eyes) and tPRK (44 eyes)	bIOP was the least affected parameter postoperatively. Among the three refractive procedures, tPRK showed the smallest reduction in IOP, SMILE an intermediate reduction and FS-LASIK the greatest
Chen et al. [18]	Prospective cohort	GAT, Corvis ST	30	35.6	50%	50 eyes with myopia <10D and/or myopic astigmatism <5D which underwent FS-LASIK (20 eyes) or SMILE (30 eyes)	bIOP did not significantly change postoperatively in both groups. GAT measurements were significantly correlated with corneal thickness after both LASIK and SMILE
Dou et al. [19]	Retrospective cohort	ORA	36	24.6	58%	71 myopic eyes with or without astigmatism who underwent either LASEK (35 eyes) or SMILE (36 eyes)	Postoperative IOPcc significantly decreased after refractive surgery. Both LASEK and SMILE affect corneal biomechanics, though SMILE appears to better preserve corneal integrity, likely due to anterior stroma preservation
Fernandez et al. [20]	Retrospective cohort	Corvis ST	43	31.6	42%	43 eyes with a sphericalequivalent (SE) from − 1.00 to − 7.00D and refractiveastigmatism below 2.25 D	bIOP remained stable after SMILE, unlike conventional IOP measurements. SMILE also significantly altered dynamic corneal parameters, and the higher incidence of an inclined brightness fringe may reflect changes in corneal biomechanics and hydration
Fu et al. [21]	Prospective cohort	Corvis ST	45	29.7	NA	91 eyes with myopia ≤ − 10 D and/or astigmatism ≤ − 5 D divided into Thin Cornea Group (46 eyes with CCT <500 μm) and Normal Cornea Group (45 eyes, CCT>500 μm)	CH, CRF, and bIOP significantly decreased after surgery in both groups. Changes in CH and CRF correlated with CCT, while greater bIOP reduction in the thin cornea group correlated with CCT
Gao et al. [22]	Prospective cohort	Corvis ST	253	24.0	53%	253 eyes with myopia of > − 0.50 D and less than − 8 D and/or astigmatism less than − 5 D	IOP and bIOP marked a significant reduction after SMILE with these values not showing association withcorneal biomechanics
Han et al. [23]	Prospective cohort	GAT, ORA	104	24.3	42%	104 eyes myopia with a spherical equivalent (SE) less than 12.50 D)	Both GAT-IOP and IOPcc significantly decreased after SMILE, though IOPcc was less affected by biomechanical changes, supporting its reliability for postoperative IOP assessment. Predictive models explained a substantial proportion of postoperative IOP variability
Hosny et al. [12]	Prospective cohort	GAT, ORA	30	29.2	53%	30 eyes with myopia and/or myopic astigmatism	GAT-IOP and IOPcc both decreased after SMILE, with a greater reduction in GAT-IOP. CH and CRF also significantly declined, and their changes were positively correlated with lenticule thickness
Joshi et al. [24]	Prospective cohort	Corvis ST	40	NA	68%	80 eyes with mild-to-moderate myopia and/or myopic astigmatism (< − 5 D spherical equivalent (SE)) divided to 2 groups (40 eyes underwent SMILE and 40 eyes underwent PRK)	bIOP decreased after surgery in both groups, with no significant postoperative difference between them. However, PRK corneas showed greater postoperative stiffness, suggesting better preservation of corneal integrity than SMILE
Li et al. [13]	Retrospective cohort	ORA	97	25.0	51%	193 eyes divided into two groups based on the refractive procedure they underwent (97 eyes in the SMILE group and 96 eyes in the FS-LASIK group)	Postoperative IOP was underestimated in both groups, though it remained more stable after SMILE. This underestimation correlated with corneal biomechanics, preoperative IOP, and flat keratometry
Li et al. [25]	Prospective cohort	ORA	202	27.4	39%	404 eyes with myopia equally divided into two groups, SMILE (202 eyes) and FS-LASIK (202 eyes)	IOP, CH, and CRF significantly decreased after both procedures, though SMILE had a smaller impact on corneal biomechanics than FS-LASIK
Liu et al. [26]	Prospective cohort	Corvis ST	40	22.9	60%	40 myopic eyes of 20 patients with SE between − 3 and − 8D and astigmatism < − 1.5D. One eye of every patient was assigned to undergo SMILE procedure with 110 μm cap thickness and the other with 150 μm	Both cap thickness groups were safe and effective, though the 110 μm group showed greater corneal spherical aberration and corrected IOP at 3 months postoperatively
Osman et al. [27]	Retrospective cohort	Corvis ST, ORA	25	26.2	48%	50 myopic eyes with or without astigmatism < 2 D equally divided into 2 groups (SMILE or FS-LASIK)	Postoperative IOP was similar between groups, while CH and CRF significantly decreased in both. However, FS-LASIK caused a much greater increase in deformation amplitude than SMILE, indicating greater biomechanical alteration
Sefat et al. [28]	Prospective cohort	GAT, Corvis ST	80	36.0	50%	128 eyes with myopia less than 10 D and/or astigmatism less than 5 D. 48 eyes underwent FS-LASIK, while 80 eyes underwent SMILE	Most Corvis ST parameters changed significantly after surgery in both groups, but matched pair analysis showed no significant biomechanical differences between them
Yu et al. [29]	Prospective cohort	ORA	32	23.4	53%	32 myopic eyes underwent LASEK and 32 myopic eyes underwent SMILE	At 3 months, CH, CRF, and IOP significantly decreased in both groups, with greater early biomechanical changes in the LASEK group. However, long-term corneal biomechanical effects were comparable between groups

bIOP, Biomechanically corrected intraocular pressure; CBI, Corvis biomechanical index; CCT, Central corneal thickness; CH, Corneal hysteresis; Corvis ST, Corneal visualization scheimpflug technology; CRF, Corneal resistance factor; DA ratio, Deformation amplitude ratio; FS-LASIK, Femtosecond Laser-assisted in situ keratomileusis; GAT, Goldmann applanation tonometry; HC deformation amp., Highest concavity deformation amplitude; IOP, Intraocular pressure; IOPcc, Corneal compensated intraocular pressure; IOPGAT/GAT-IOP, Goldmann applanation tonometry intraocular pressure; IOPNCT, Non-contact tonometry intraocular pressure; IR, Integrated radius; LASEK, Laser-assisted subepithelial keratectomy; ORA, Ocular response analyzer; PRK/tPRK, (Transepithelial) Photorefractive Keratectomy; RST index, Relative stromal thickness index; SE, Spherical equivalent; SMILE, Small incision lenticule extraction; SP-A1, Stiffness parameter at first applanation; SSI, Stress–strain index

### Postoperative changes in intraocular pressure

#### Goldmann applanation tonometer (GAT)

**Fig. 2:**
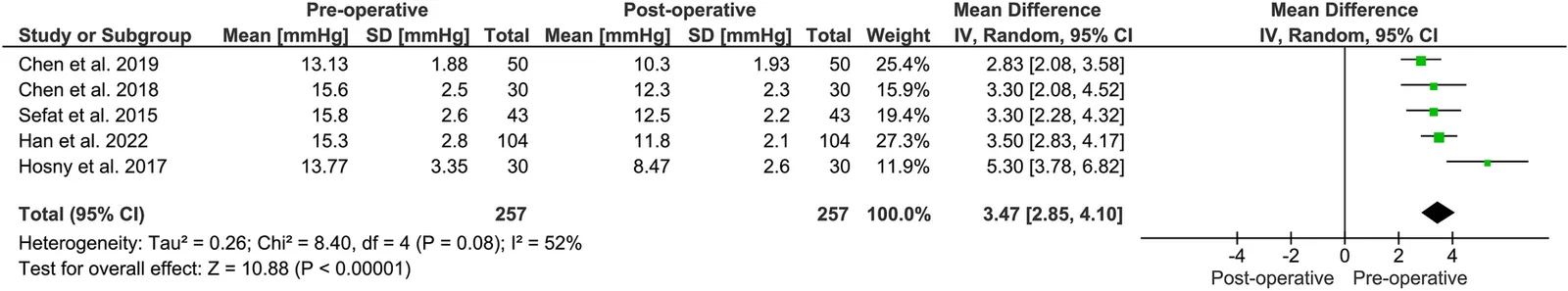
Forest plot of the estimated mean difference (MD) in intraocular pressure (IOP) measured with GAT before (pre-operative) and after (post-operative) SMILE. A random-effects model was applied; SD, standard deviation; CI, confidence interval

In the present analysis, 5 studies were included, examining a total of 257 eyes before and after SMILE. The meta-analysis revealed a statistically significant decrease in IOP measured with GAT, with an estimated mean difference of 3.47 mmHg (95% CI 2.85–4.10,* p* < 0.001). Moderate heterogeneity was observed among the included studies, with an I^2^ value of 52% (Fig. 2).

#### Ocular response analyzer (ORA).

**Fig. 3:**
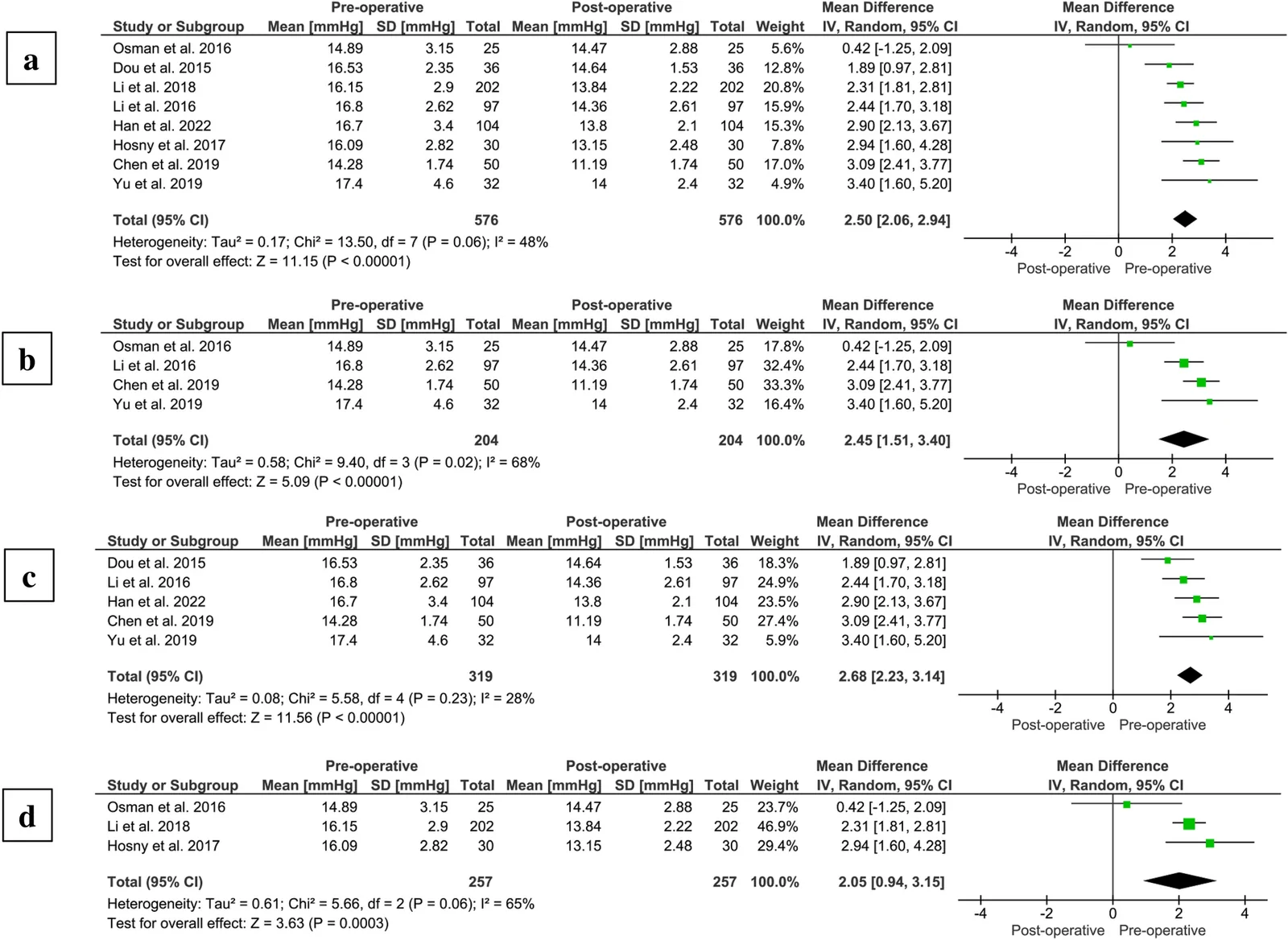
Forest plots showing the pooled mean difference (MD) in intraocular pressure (IOP) measured with the Ocular Response Analyzer (ORA) before (preoperative) and after (postoperative) SMILE. **a** Overall analysis including all eligible studies; **b** sensitivity analysis including only studies reporting both the number of patients and eyes to address the unit-of-analysis issue; **c** subgroup analysis of studies with a follow-up duration of ≥3 months; **d** subgroup analysis of studies with a follow-up duration of < 3 months. A random-effects model was used for all analyses. MD, mean difference; CI, confidence interval; SD, standard deviation

A total of 8 studies were included in this analysis, encompassing 576 eyes assessed before and after SMILE. The meta-analysis revealed a statistically significant decrease in IOP measured with ORA, with an estimated mean difference of 2.50 mmHg (95% CI 2.06–2.94,* p* <0.001). Low heterogeneity was observed among the included studies, with an I^2^ value of 48% (Fig. 3a).

To address the unit-of-analysis, 4 studies reporting both the number of patients and eyes were included, comprising 102 patients (204 eyes) evaluated before and after SMILE. The meta-analysis demonstrated a statistically significant decrease in IOP measured with ORA, with an estimated mean difference of 2.45 mmHg (95% CI 1.51–3.40, * p* < 0.001). Moderate heterogeneity was observed among the included studies (I^2^ = 68%) (Fig. 3b). A subgroup analysis including studies with a follow-up duration of ≥ 3 months was performed. Five studies comprising 319 eyes were included. The meta-analysis demonstrated a statistically significant reduction in IOP measured with ORA, with a pooled mean difference of 2.68 mmHg (95% CI 2.23–3.14,* p* < 0.001). Low heterogeneity was observed among the included studies, with an I^2^ value of 28% (Fig. 3c). Three studies comprising 257 eyes with a follow-up of <3 months were included. ORA-measured IOP was significantly reduced after SMILE (MD: 2.05 mmHg, 95% CI 0.94–3.15; * p* = 0.0003), with moderate heterogeneity (I^2^ = 65%) (Fig. 3d).

#### Corneal visualization scheimpflug technology (Corvis ST)

**Fig. 4:**
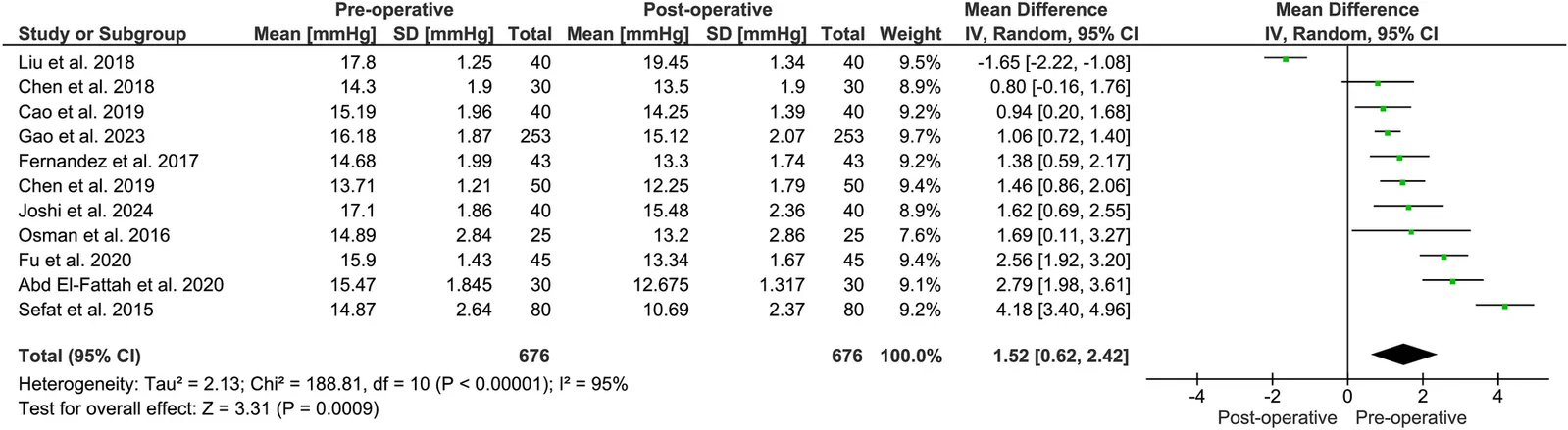
Forest plot of the estimated mean difference (MD) in intraocular pressure (IOP) measured with Corvis ST before (pre-operative) and after (post-operative) SMILE. A random-effects model was applied; SD, standard deviation; CI, confidence interval

**Fig. 5:**
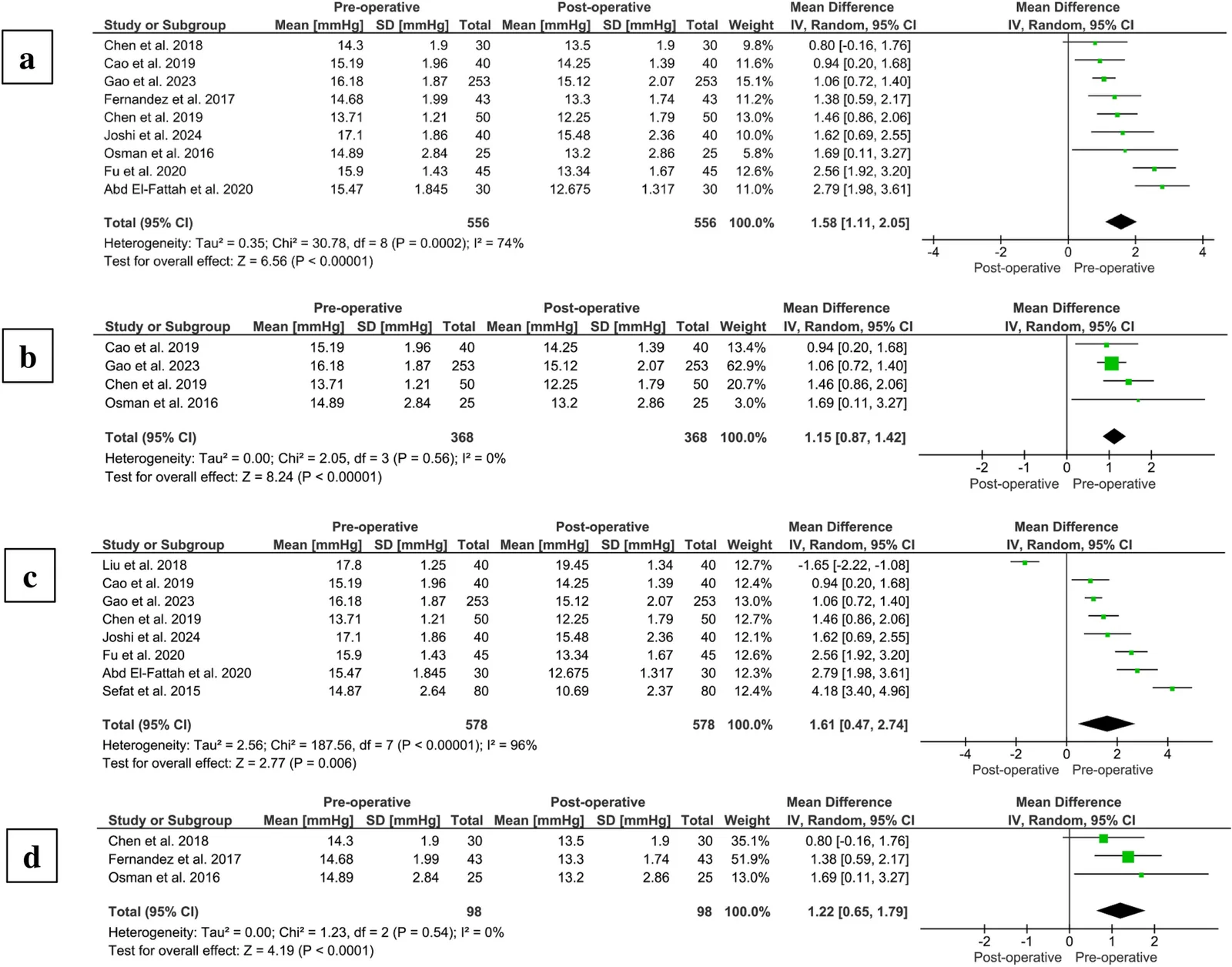
Forest plots showing the pooled mean difference (MD) in intraocular pressure (IOP) measured with Corvis ST before (preoperative) and after (postoperative) SMILE. **a** Sensitivity analysis excluding two studies following funnel plot evaluation; **b** subgroup analysis including only studies reporting both the number of patients and eyes to address the unit-of-analysis issue; **c** subgroup analysis of studies with a follow-up duration of ≥3 months; **d** subgroup analysis of studies with a follow-up duration of < 3 months. A random-effects model was used for all analyses. MD, mean difference; CI, confidence interval; SD, standard deviation

In this analysis, 11 studies were included, comprising a total of 676 eyes evaluated before and after SMILE. The meta-analysis demonstrated a statistically significant reduction in IOP measured with Corvis ST, with a calculated mean difference of 1.52 mmHg (95% CI 0.62–2.42, * p* < 0.001). The heterogeneity among the included studies was substantial, with an I^2^ value of 95%. Egger’s regression test did not indicate significant publication bias (intercept = − 3.13,* p* = 0.417) (Fig. 4). A sensitivity analysis was performed by excluding two studies after funnel plot evaluation, resulting in 9 studies comprising 556 eyes evaluated before and after SMILE. The analysis continued to demonstrate a statistically significant reduction in IOP measured with Corvis ST, with a mean difference of 1.58 mmHg (95% CI 1.11–2.05, * p* < 0.001). Notably, heterogeneity was moderate compared to the primary analysis, with an I^2^ value of 74%, indicating improved consistency among the included studies (Fig. 5a).

To evaluate the potential impact of the unit-of-analysis, a subgroup analysis including four studies comprising 184 patients (368 eyes) was performed. Corvis ST-measured IOP remained significantly lower after SMILE (MD: 1.15 mmHg, 95% CI 0.87–1.42; *p* < 0.001), with no observed heterogeneity (I^2^ = 0%) (Fig. 5b). In the subgroup of studies with a follow-up duration of ≥3 months, Corvis ST-measured IOP remained significantly reduced after SMILE (8 studies, 578 eyes; MD: 1.61 mmHg, 95% CI 0.47–2.74; *p* = 0.006), despite substantial heterogeneity (I^2^ = 96%) (Fig. 5c). In the subgroup of studies with a follow-up duration of < 3 months, Corvis ST-measured IOP was significantly reduced after SMILE (3 studies, 98 eyes; MD: 1.22 mmHg, 95% CI 0.65–1.79; *p* < 0.001), with no observed heterogeneity (I^2^ = 0%) (Fig. 5d).

### Postoperative changes in corneal biomechanics using ocular response analyzer (ORA)

#### Corneal hysteresis (CH)

**Fig. 6:**
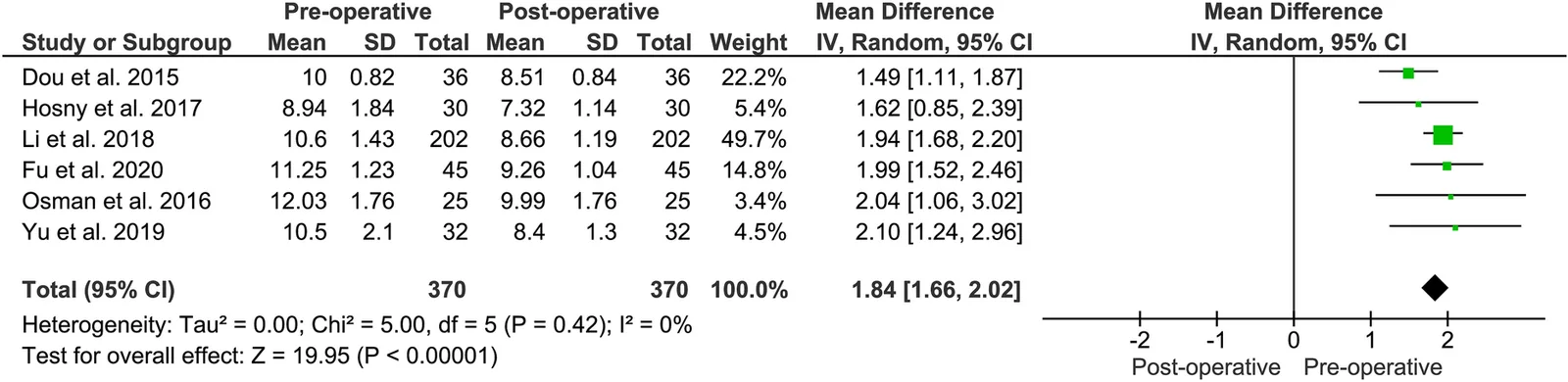
Forest plot of the estimated mean difference (MD) in corneal hysteresis (CH) measured with ORA before (pre-operative) and after (post-operative) SMILE. A random-effects model was applied; SD, standard deviation; CI, confidence interval

The meta-analysis demonstrated a significant reduction in corneal hysteresis (CH) following refractive surgery across all included studies. The pooled analysis included 6 studies of 370 eyes, showing that postoperative CH values were significantly lower compared with preoperative measurements, with a mean difference (MD) of 1.84 mmHg (95% CI 1.66 to 2.02;* p* < 0.001). Heterogeneity among studies was negligible (I^2^ = 0%, * p* = 0.42), indicating excellent consistency of findings across studies. The overall effect size was highly significant (* p* < 0.001) (Fig. 6).

#### Corneal resistance factor (CRF)

**Fig. 7:**
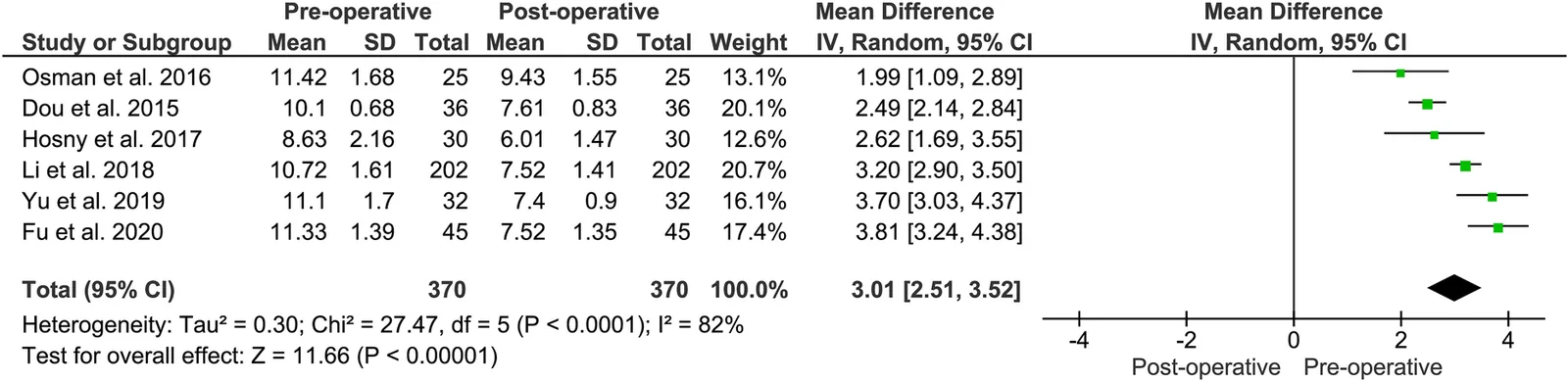
Forest plot of the estimated mean difference (MD) in corneal resistance factor (CRF) measured with ORA before (pre-operative) and after (post-operative) SMILE. A random-effects model was applied; SD, standard deviation; CI, confidence interval

In this analysis, 6 studies were included, comprising a total of 370 eyes evaluated before and after SMILE. The meta-analysis revealed that postoperative CRF values were significantly lower compared with preoperative measurements, with a mean difference (MD) of 3.01 mmHg (95% CI 2.51 to 3.52; * p* < 0.001). Substantial heterogeneity was observed among studies (I^2^ = 82%,* p* < 0.01), indicating substantial variability in effect sizes across the included studies. Nevertheless, the overall pooled effect remained highly significant (* p* < 0.001) (Fig. 7).

## Discussion

This systematic review and meta-analysis provide a comprehensive evaluation of intraocular pressure (IOP) measurements and corneal biomechanical parameters in myopic eyes and eyes with myopic astigmatism after SMILE refractive surgery. Our findings indicate a significant postoperative reduction in measured IOP across all tonometric modalities assessed, alongside a significant reduction in corneal viscoelastic parameters, specifically Corneal Hysteresis (CH) and Corneal Resistance Factor (CRF).

Assessment of IOP is a pivotal component of post refractive surgery follow-up, particularly in patients where precise IOP measurement is required. Accuracy of IOP evaluation in patients who subsequently develop ocular hypertension or glaucoma is especially important, since postoperative underestimation may delay diagnosis, influence target IOP determination, and complicate longitudinal disease monitoring. According to the results of this meta-analysis, postoperative reductions in IOP measurements varied across tonometric devices, with GAT exhibiting the largest decrease in measured IOP (MD: 3.47 mmHg), followed by the ORA (MD: 2.50 mmHg) and Corvis ST (MD: 1.52 mmHg). Importantly, although GAT remains the gold standard method for IOP assessment, it appears to significantly underestimate IOP measurements following SMILE surgery. GAT measurements are highly dependent on corneal thickness and biomechanical properties [12]. As none of the included studies directly quantified intracameral IOP, the observed postoperative reductions should be interpreted as changes in tonometrically measured IOP resulting from the combined influence of intraocular pressure and SMILE-induced alterations in corneal thickness and biomechanical properties, rather than as direct evidence of true IOP underestimation. The magnitude of reduction correlates with lenticule thickness—thicker lenticules (higher myopic corrections) produce greater decreases both in CH and CRF resulting in reduced corneal viscoelastic damping capacity and overall corneal rigidity [19]. Several studies have demonstrated that greater lenticule thickness and stromal tissue removal are significantly associated with larger reductions in CH and CRF, indicating more pronounced postoperative biomechanical weakening [19,30,31]. Although CH and CRF are commonly used to characterize corneal biomechanical behavior, they represent indirect indices rather than direct measurements of intrinsic material properties, such as corneal stiffness or Young's modulus. Accordingly, the observed postoperative reductions should be interpreted as reflecting biomechanical changes rather than providing direct evidence of biomechanical weakening [32,33]. Postoperative CRF and CH have been identified as significant predictors of reduction in measured IOP after SMILE, supporting the concept that postoperative biomechanical weakening contributes directly to tonometric measurement error [13].

ORA-derived IOP measurements also demonstrated significant postoperative reductions, despite IOPcc being specifically designed to account for corneal biomechanical properties [11]. Despite our findings were evaluated based on IOPcc, IOP underestimation was statistically significant using a large number of eyes underwent SMILE refractive surgery (576 eyes). This shows that ORA's IOPcc algorithm, while partially compensating for corneal properties, does not fully correct for the biomechanical changes induced by lenticule extraction. Predictive models incorporating preoperative IOP, postoperative CRF, CH, and flat keratometry can help estimate true IOP [23]. Corvis ST measurements showed the smallest postoperative reduction in IOP among all included devices. Importantly, the smaller postoperative reduction in IOP observed with ORA and particularly Corvis ST should not be interpreted as reflecting a smaller true change in IOP. Rather, it most likely reflects the biomechanical compensation algorithms incorporated into these devices, which are designed to reduce the influence of corneal biomechanical alterations on IOP measurements following refractive surgery [34,35]. After SMILE, Corvis ST dynamic corneal response (DCR) parameters indicate reduced corneal biomechanical stiffness. Specifically, deformation amplitude (DA) and integrated radius (IR) increase, reflecting greater corneal deformability, while stiffness parameter at first applanation (SP-A1) decreases, indicating reduced corneal stiffness. Additionally, first and second applanation times (A1T and A2T) are significantly altered, consistent with a softer postoperative cornea [36,37]. The bIOP algorithm on the Corvis ST accounts for corneal geometry and biomechanical properties and performs substantially better post-SMILE [38]. The latest algorithms (bIOP v2) have been clinically validated to be independent of age and corneal thickness, showing a median change of less than 1 mmHg after surgery and demonstrating superior stability compared to older correction versions [18]. Several studies have shown that bIOP remains largely unaffected after SMILE, showing only minimal and non-significant changes of approximately 0.6–0.8 mmHg postoperatively [18]. However, our pooled findings indicate that SMILE causes a small but statistically significant underestimation of IOP even when measured with Corvis ST’s biomechanically corrected IOP (bIOP) algorithm. Numerically, the largest postoperative reductions were observed with GAT, followed by ORA and Corvis ST; however, no formal indirect comparison was performed between devices, and these differences may also reflect heterogeneity in the populations contributing to each subgroup. Sensitivity and subgroup analyses were performed to investigate the substantial heterogeneity observed in the primary Corvis ST analysis. The findings suggest that methodological and clinical differences among studies contributed to the observed variability.

An important distinction must be drawn between statistical and clinical significance. The European Glaucoma Society identifies a minimal clinically important difference (MCID) of 2–3 mmHg for IOP when evaluating new glaucoma surgical devices [9], a framework also endorsed by the American Academy of Ophthalmology Glaucoma Preferred Practice Pattern Committee [39]. Applied to our findings, the GAT reduction (3.47 mmHg) exceeds this threshold and is therefore clinically significant; the ORA–IOPcc reduction (2.50 mmHg) is borderline; and the Corvis ST–bIOP reduction (1.52 mmHg) lies below the MCID, indicating that the bIOP algorithm partially compensates for SMILE-induced biomechanical changes. However, sub-MCID biases are not necessarily inconsequential in glaucoma-relevant populations: each 1 mmHg of IOP corresponds to approximately a 10% change in glaucoma progression risk [40], so even small residual underestimations may influence target-IOP setting and progression monitoring in established or suspected glaucoma.

### Limitations

All included studies were observational (prospective or retrospective cohorts); no randomized controlled trials were available, and the absence of contemporaneous control eyes precluded adjustment for natural fluctuation in IOP. All included studies enrolled healthy myopic individuals undergoing SMILE, the findings cannot be directly generalized to patients with glaucoma or ocular hypertension. Moreover, substantial heterogeneity was present for the Corvis ST analysis (I^2^ = 95%) and the CRF analysis (I^2^ = 82%), reflecting differences in patient populations, lenticule thickness, refractive error, follow-up timing. Importantly, the timing of intraocular pressure measurements was not consistently standardized or reported across the included studies, and therefore the potential influence of diurnal intraocular pressure variation on the pooled estimates cannot be excluded. Additionally, the inclusion of both eyes without adjustment for inter-eye correlation in some studies may have introduced unit-of-analysis bias. Finally, our pooled biomechanical analyses were limited to corneal hysteresis and corneal resistance factor; the broader set of Corvis ST dynamic Scheimpflug parameters (e.g., DA Ratio, integrated inverse radius, SP-A1, CBI, SSI) could not be meta-analysed because of inconsistent reporting across studies and should be addressed by future research, ideally using multivariate or machine-learning approaches.

## Conclusions

SMILE surgery leads to a significant postoperative reduction in measured IOP across all tonometers, with GAT showing the largest underestimation, followed by ORA and Corvis ST. These changes are accompanied by significant reductions in clinically relevant corneal biomechanical parameters, such as CH and CRF. Biomechanical parameters produced by these devices can be used to quantify the reduction of measured IOP after SMILE and may be incorporated into novel corrective algorithms for postoperative IOP assessment. Clinicians should be aware that even biomechanically corrected IOP readings may underestimate true IOP post-SMILE, warranting caution in glaucoma screening and monitoring. The three tonometric modalities should not be considered interchangeable in post-SMILE eyes, as the magnitude of postoperative measurement change differs substantially between devices and clinical decisions based on one device’s reading may not translate directly to another. Future studies directly comparing tonometric devices in the same post-SMILE eyes, with adjustment for population heterogeneity, would help establish device-specific correction factors and clarify which modality provides the most reliable IOP measurement after refractive surgery.

## Supplementary Information

Below is the link to the electronic supplementary material.

Supplementary file1 (DOCX 23 KB)

## Data Availability

No datasets were generated or analysed during the current study.
