# Supplementary material for: Effects of SMILE on intraocular pressure and corneal biomechanics: a systematic review and meta-analysis
Source: Int Ophthalmol. 2026 Aug 24;46(1):342. doi: 10.1007/s10792-026-04194-5 (PMC13503405; doi:10.1007/s10792-026-04194-5)
Supplement: Supplementary file 1 [file 10792_2026_4194_MOESM1_ESM.docx]

***Supplementary Material***

**Supplementary Table S1.** Search queries regarding the changes of Intraocular pressure before and after Small incision lenticule extraction (SMILE) surgery.

| **Database** | **Query** | **Results** |
| --- | --- | --- |
| **Pubmed** | (SMILE OR "small incision lenticule extraction") AND ("intraocular pressure" OR IOP) | 114 |
| **Scopus** | TITLE-ABS-KEY (SMILE OR "small incision lenticule extraction") AND ("intraocular pressure" OR IOP) | 281 |
| **ScienceDirect** | (SMILE OR "small incision lenticule extraction") AND ("intraocular pressure" OR IOP) | 384 |
| **Cochrane Library** | (SMILE OR "small incision lenticule extraction") AND ("intraocular pressure" OR IOP) | 28 |

**Supplementary Table S2.** Quality and Risk-of-Bias assessment using the Newcastle-Ottawa Scale (NOS)

| **Study** | **Selection**  **0–4** | **Comparability**  **0–2** | **Outcome**  **0–3** | **Total stars**  **0–9** | **Quality** | **Comments** |
| --- | --- | --- | --- | --- | --- | --- |
| Abd El-Fattah et al. 2020 | 4 | 2 | 2 | 8 | Good | Strict inclusion/exclusion criteria and 6-month follow-up; Outcome technician was masked but both eyes from the same patient were used. |
| Cao et al. 2019 | 4 | 1 | 3 | 8 | Good | One eye per patient used; clear criteria and Corvis ST follow-up to 3 months; Comparability limited because allocation was by patient willingness |
| Chen et al. 2019 | 4 | 2 | 3 | 9 | Good | Matched groups; One eye per patient used; 3 months follow-up |
| Chen et al. 2018 | 3 | 1 | 2 | 6 | Fair | Clear SMILE/LASIK evaluation and validated GAT/bIOP measurements; some patients contributed both eyes and comparability was limited |
| Dou et al. 2015 | 4 | 1 | 2 | 7 | Good | Clear inclusion/exclusion criteria; Possibility of bias because of 36 eyes from 21 SMILE patients |
| Fernandez et al. 2017 | 3 | 0 | 2 | 5 | Fair | Validated Corvis outcomes and 1 month follow-up but no comparator and several cases were excluded as outliers/erroneous measurements |
| Fu et al. 2020 | 3 | 0 | 3 | 6 | Fair | Only the normal cornea control group was used for data extraction; because only one-arm was used for the meta-analysis comparability was not applicable |
| Gao et al. 2023 | 4 | 0 | 3 | 7 | Good | Large single-arm study; 253 eyes from 253 patients, only right eye selected to prevent inter-eye correlation. No comparator group. |
| Han et al. 2022 | 4 | 0 | 3 | 7 | Good | Clear eligibility criteria, 6 months follow-up. No comparator group. |
| Hosny et al. 2017 | 3 | 0 | 2 | 5 | Fair | Both eyes from the same patients were included and no correction for inter-eye correlation was applied. |
| Joshi et al. 2024 | 4 | 2 | 2 | 8 | Good | Clear eligibility criteria, 6-month follow-up. No blinding and both eyes from the same patient included. |
| Li et al. 2016 | 4 | 1 | 2 | 7 | Good | 193 eyes from 193 patients; Clear criteria; Attrition at 6 months was substantial with fewer eyes completing long-term follow-up. |
| Li et al. 2018 | 1 | 1 | 1 | 3 | Poor | Data extracted from English abstract only; Numerical ORA data were available but full assessment of methods, selection, follow-up completeness, and bias is limited. |
| Liu et al. 2018 | 4 | 2 | 3 | 9 | Good | Randomization by eye, clear criteria, trial registration and 3-month assessment. |
| Osman et al. 2016 | 3 | 1 | 2 | 6 | Fair | Retrospective study; ORA/Corvis ST outcomes and 1-month follow-up required; Selection and completeness of follow-up are less robust than in prospective studies. |
| Sefat et al. 2015 | 3 | 1 | 2 | 6 | Fair | Validated GAT/Corvis ST measurements but baseline SE and tissue removal differed between groups, requiring matched subgroup analysis. |
| Yu et al. 2019 | 4 | 1 | 3 | 8 | Good | Right eye only analyzed,3-month and 3-year follow-up, all patients completed examinations. For the present meta-analysis, 3-month postoperative measurements were extracted to ensure consistency with the follow-up period of other included studies. |
| bIOP, biomechanically corrected intraocular pressure; Corvis ST, Corneal Visualization Scheimpflug Technology; GAT, Goldmann applanation tonometry; LASIK, laser-assisted in situ keratomileusis; ORA, Ocular Response Analyzer; SE, spherical equivalent, SMILE; small incision lenticule extraction | | | | | | |
